# Brief overview of dietary intake, some types of gut microbiota, metabolic markers and research opportunities in sample of Egyptian women

**DOI:** 10.1038/s41598-022-21056-z

**Published:** 2022-10-14

**Authors:** Nayera E. Hassan, Salwa M. El Shebini, Sahar A. El-Masry, Nihad H. Ahmed, Ayat N. Kamal, Ahmed S. Ismail, Khadija M. Alian, Mohammed I. Mostafa, Mohamed Selim, Mahmoud A. S. Afify

**Affiliations:** 1 Biological Anthropology Department, Medical Research and Clinical Studies Institute, National Research Centre, 33 El-Buhouth St., Dokki, Giza, 12622 Egypt; 2 Nutrition and Food Science Department, Nutrition and Food Science Institute, National Research Centre, Giza, Egypt; 3 Clinical Pathology Department, Medical Research and Clinical Studies Institute, National Research Centre, Giza, Egypt; 4 Researches and Applications of Complementary Medicine Department, Medical Research and Clinical Studies Institute, National Research Centre, Giza, Egypt

**Keywords:** Gastroenterology, Medical research

## Abstract

Metabolic syndrome (MetS) is a phenotype caused by the interaction of host intrinsic factors such as genetics and gut microbiome, and extrinsic factors such as diet and lifestyle. To demonstrate the interplay of intestinal microbiota with obesity, MetS markers, and some dietary ingredients among samples of Egyptian women. This study was a cross-sectional one that included 115 Egyptian women; 82 were obese (59 without MetS and 23 with MetS) and 33 were normal weight. All participants were subjected to anthropometric assessment, 24 h dietary recall, laboratory evaluation of liver enzymes (AST and ALT), leptin, short chain fatty acids (SCFA), C-reactive protein, fasting blood glucose, insulin, and lipid profile, in addition to fecal microbiota analysis for Lactobacillus, Bifidobacteria, Firmicutes, and Bacteroid. Data showed that the obese women with MetS had the highest significant values of the anthropometric and the biochemical parameters. Obese MetS women consumed a diet high in calories, protein, fat, and carbohydrate, and low in fiber and micronutrients. The Bacteroidetes and Firmicutes were the abundant bacteria among the different gut microbiota, with low Firmicutes/Bacteroidetes ratio, and insignificant differences between the obese with and without MetS and normal weight women were reported. Firmicutes/Bacteroidetes ratio significantly correlated positively with total cholesterol and LDL-C and negatively with SCFA among obese women with MetS. Findings of this study revealed that dietary factors, dysbiosis, and the metabolic product short chain fatty acids have been implicated in causing metabolic defects.

## Introduction

Metabolic syndrome (MetS), differently known as insulin resistance, syndrome X, etc.., is characterized by WHO as a pathologic condition characterized by abdominal obesity, hyperleptinemia, hypertension, and insulin resistance^1^. In spite of the fact that there's some variety within the definition by other wellbeing care organization, the contrasts are minor. With the effective success in approximately elimination of communicable infectious diseases in most of the world, this modern non-communicable disease (NCD) has gotten to be the major health hazard of advanced world. The two fundamental forces spreading this ailment are the increment intake of high calorie and low fiber fast food and diminish in physical activity due to mechanized transportations and sedentary frame of leisure time exercises^2^.

Recent clinical and experimental research revealed that the gut microbiota is one of the foremost vital pathogenic variables in MetS^3^. Metabolic syndrome itself could be a phenotype caused by the interaction of host intrinsic variables such as genetics and the gut microbiome, and extrinsic components such as diets and the way of life. Metabolic syndrome is frequently accompanied by an imbalance of the gut microbiota, causes a low-grade inflammatory reaction within the body by wrecking the intestine barrier, creating insulin resistance through metabolites influencing host metabolism and hormone secretion, shaping a vicious circle that advances the persistent progress of MetS. Subsequently, intestinal microbiota may be a potential target for the treatment of MetS^4^.

Trillions of microorganisms live in symbiosis within the human body, and are primarily found within the gastrointestinal system, oral mucosa, saliva, skin, conjunctiva, and vagina^5^. The number of the microorganisms; that occupy the gastrointestinal tract (i.e., gut microbiota); be around 1 × 1014^6^ and play a basic role in intestinal homeostasis, development, and protection against pathogens. In addition, their presence within the intestine is related to immunomodulatory and metabolic responses^7^.

Gut microbiota comprises of microbes, yeasts, and viruses. Intestine bacteria are more than 1000 species that have related to six overwhelming phyla: Firmicutes, Bacteroidetes, Actinobacteria, Proteobacteria, Fusobacteria, and Verrucomicrobia. The phyla Firmicutes and Bacteroidetes are the foremost common bacteria representing 90% of the gut microbiota^8^.

Firmicutes bacteria are Gram-positive one. It plays a key role in the nutrition and metabolism of the host through SCFA synthesis. Through their metabolic products, Firmicutes bacteria are indirectly connected with other tissues and organs and regulate hunger and satiety. In contrast, Bacteroidetes bacteria are Gram-negative and associated with immunomodulation. Their components, lipo-poly-saccharides and flagellin, interact with cell receptors and enhance immune reactions through cytokine synthesis. Increased or decreased Firmicutes/Bacteroidetes (F/B) ratio are associated with the development of obesity or irritable bowel disease (IBD)^9^.

Firmicutes, due to their negative influence on glucose and fat metabolism, are commonly referred to as bad gut microbes^10^. Accumulating evidence proved that short chain fatty acids (SCFA) play a critical role in supporting the intestine and metabolic health. The SCFA acetic acid, propionate and butyrate are the main metabolites produced by the microbiota in the large intestine through the anaerobic fermentation of indigestible carbohydrates; they play a crucial role to gastrointestinal health^11^.

Microbial SCFA generation is basic for intestine integrity by controlling the luminal pH and mucus production. They are the main energy source for epithelial cells and speculated to play a key role in mucosal immune function. SCFA moreover straight forwardly modulate the metabolic health of the host through multiple neurochemical pathways related to energy expenditure, glucose homeostasis, appetite regulation, and immune-modulation^12^.

This study aimed to identify the interplay of the intestinal microbiota with obesity, metabolic markers and some dietary ingredient; especially the fat content; among a sample of Egyptian women.

## Subjects and methods

A cross-sectional study included 115 Egyptian women, with ages ranged between 25 and 60 years; mean age 41.62 ± 10.70 years. They were recruited and randomly chosen, from all employees and workers; of all categories; of the “National Research Centre”, Egypt. A written informed consent was obtained from all participants after being informed about the purpose of the study. This research paper was derived from a cross-sectional survey of a project funded by National Research Centre, Egypt, 2019–2022 entitled ‘‘Gut Microbiota in Obesity and Metabolic syndrome among obese women: Interactions of the Microbiome, Epigenetic, Nutrition and Probiotic Intervention.” (12th Research Plan of the National Research Centre), which was approved from “Ethics Committee of National Research Centre” (Registration Number is19/236). All used methods were performed in accordance with the relevant guidelines and regulations.

## Methods

For each participated woman, blood pressure, anthropometric measurements, 24 h dietary recall, laboratory investigations and microbiota analysis were done.

### Blood pressure

Blood pressure was measured using the standardized mercury sphygmomanometer with a suitable cuff size. It was measured on the left arm while the participated women were sitting relaxed for 5 min. Two readings were obtained and the average was recorded. Systolic blood pressure (SBP); determined by the onset of the “tapping” korotkoff sounds (K1), while the fifth korotkoff sound (K5), or the disappearance of korotkoff sounds, as the definition of diastolic blood pressure (DBP) were recorded.

### Anthropometric measurements

Body weight, height, neck, hip and waist circumferences were measured, following the recommendations of the “International Biological Program”^13^. Body weight (Wt) was determined to the nearest 0.01 kg using a Seca Scale Balance, with the woman wearing minimal clothes and with no shoes. Body height (Ht) was measured to the nearest 0.1 cm using a Holtain portable anthropometer. Circumferences was measured using non-stretchable plastic tape; approximated to the nearest 0.1 cm. Neck Circumference was measured at a point mid-way between the collarbone and chin in the middle of the neck while Standing or sitting with a straight back. Waist circumference (WC) was measured at the midpoint between the lower curvature of the last fixed rib and the superior curvature of the iliac crest, with the woman in an upright standing position and their arms alongside the body, feet together, and abdomen relaxed. Hip circumference was measured at the maximum extension of the buttocks measuring the largest diameter above the symphisis pubis overlapping the apex of the buttocks. Waist/hip ratio [WC/Hip C in cm] and Body mass index (BMI) [BMI: weight (in kilograms) divided by height (in meters squared)] were calculated. The participated women were all chosen as obese; as their BMI ≥ 30 kg/m^2^.The participant women were classified according to their BMI into 2 groups: 30 women with normal BMI (BMI = 18– < 25 kg/m^2^) and 82 obese women (BMI ≥ 30 kg/m^2^).

### Dietary recalls

Information from each participant about her usual pattern of food intake was obtained. Data was collected by means of dietary interview consisting of 24 h recall that repeated for 3 days, and a food frequency questionnaire. Analysis of food items was done using World Food Dietary Assessment System, (WFDAS), USA, University of California^14^.

### Blood sampling and laboratory investigations

In the morning, venous blood samples (after 12-h fasting) were drawn from the participated women, using venipuncture. Biochemical parameters were performed on fasting sera that were stored at – 70 °C until used for assessment of liver enzymes: Aspartate amino-transferase (AST) and Alanine amino-transferase (ALT), leptin, Short Chain Fatty Acids (SCFA), C-reactive protein (CRP), fasting blood glucose (FBG), insulin, and lipid profile. All were done in the laboratory of “Medical Excellence Research Center” which is a part of the “National Research Centre”, Egypt.

Serum concentrations of AST and ALT were determined using the automated clinical chemistry analyzer Olympus AU 400 analyzer (https://www.mybiosource.com).

The assay of human Leptin in serum was performed by ELISA method, using kits of BioLegend, Inc., (San Diego – USA), according to the method of Considine *et al*.^15^.

Human Short Chain Fatty Acids (SCFA) were assessed in serum using Enzyme Linked Immuno-sorbent Assay (ELISA) kits; Catalog Number: MBS7269061 according to the method described by den Besten et al^16^.

Fasting blood glucose (FBG) level was measured using the automated clinical chemistry analyzer Olympus AU 400 analyzer. Serum insulin was assessed using Enzyme Immunoassay Test Kit Catalog No. E29-072(Immunospec Corporation).

The assay of the serum CRP was performed by Enzyme Linked Immuno-sorbent Assay (ELISA) kits, Cat No.: RAP002^17^, (https://www.mybiosource.com.)

Estimation of lipid profile: Serum levels of total cholesterol (TC), triglycerides (TG), high density lipoprotein cholesterol (HDL-C) were measured by standardized enzymatic procedures; using kits supplied by Roche Diagnostics (Mannheim, Germany) on the Olympus AU 400 automated clinical chemistry analyzer. Low density lipoprotein cholesterol (LDL-C) was calculated according to formula of Friedewald et al.^18^ as follows: LDL – C = Total cholesterol – Triglycerides/5 + HDL-C.

Clinically, a patient is considered to have MetS when three or more of the following five conditions exist, which are (1) waist circumference ≥ 88 cm in women, (2) blood pressure ≥ 135/85 mmHg, (3) triglycerides ≥ 150 mg/dl, (4) HDL-C < 50 mg/dl in women, and (5) fasting glucose ≥ 100 mg/dl^19^.

### Microbiota analysis

The proportion of Lactobacillus and Bifidobacteria; and Firmicutes/Bacteroidetes ratio strains were assessed in the stool of all participants by using the real time PCR (Polymerase Chain Reaction). Specimen collection and preparation: Stool was collected by defecation in a plain sterilized container allowed to be frozen. Specimen Storage and Preparation: stool was frozen on at − 20°. The primers and probes were used to detect Bifidobacterium spp. and Lactobacillus spp; and Firmicutes spp. and Bacteroidetes spp., where based on 16S rRNA gene sequences retrieved from the National Center for Biotechnology Information databases by means of the Entrez program^20^. Primer sets used in this study. Target organism Primer Set Sequence (5′ to 3′) Product Size (bp) Ta (°C), time (s) Reference Lactobacillus Lacto-16S-F GGA ATC TTC CAC AAT GGA CG . genus Lacto-16S-R CGC TTT ACG CCC AAT AAA TCC GG 216 56, 10 s Bifidobacterium g-Bifid-F CTC CTG GAA ACG GGT GG Matsuki et al. (2004) genus g-Bifid-R GGT GTT CTT CCC GAT ATC TAC A 562 (549–563) 61, 20s Matsuki et al. (2004). Bacteroidetes: 798cfbF AAACTCAAAKGAATTGACGG (Forward), and cfb967R GGTAAGGTTCCTCGCGCTAT (Reverse). Firmicutes: 928F–firm TGAAACTYAAGGAATTGACG(Forward),and1040FirmR CCATGCACCACCTGTC (Reverse), and universal bacterial 16S rRNA sequences: 926F AAACTCAAAKGAATTGACGG(Forward), and 1062R CTCACRRCACGAGCTGAC (Reverse).

Reagents provided by kits: DNA extraction Kit. Assay procedure: DNA extraction: The QIAamp DNA Stool Minikit (Qiagen) was used to extract DNA from one gram of fresh or frozen stool sample according to the manufacturer's instructions. Bacterial quantification by real-time PCR was done.

### Statistical analysis

Data were analyzed using the Statistical Package for Social Sciences (SPSS/Windows Version 18, SPSS Inc., Chicago, IL, USA). Normality of data was tested using the Kolmogorov–Smirnov test. The data were normally distributed. So, the parametric tests were used.

The participated women were classified into: 33 with normal BMI (18– < 25 kg/m^2^) and 82 obese with BMI ≥ 30 kg/m^2^. They obese women were classified according to the presence of MetS markers into two groups: 59 obese without MetS (have no or less than 2 markers of MetS), and 23 obese with MetS (have 3 or more markers of MetS).

The parametric data were expressed as mean ± SE. The various parametric variables of the two groups were analyzed and compared using independent t test. Pearson’s correlation test was used to assess the relations between Firmicutes/Bacteroid ratio and the clinical and metabolic parameters, and between gut microbiota and daily intake of total fat, carbohydrate and fiber among the three groups. *p* < 0.05 was regarded as statistically significant for all tests.

## Results

Table 1 showed the mean ± SE of the age, blood pressure, anthropometric and body composition parameters of the studied sample. Data revealed highly significant difference between the three groups in most of the parameters at *p* ≤ 0.01; where the obese women with MetS had the highest values. While the obese women without MetS had the significant highest values regarding WHR, NC and FFM (*p* < 0.05).

**Table 1:** Characteristic anthropometric parameters and blood pressure of the studied women ANOVA.

Parameters	Obese		Control N = 33	*p*
	Obese without MetS N = 59	Obese with Mets N = 23		
	Mean ± SEM			
Age (year)	48.65 ± 0.85	43.47 ± 0.59	31.70 ± 0.61^b,c^	0.000**
**Blood pressure**				
SBP (mmHg)	115.38 ± 0.45	137.50 ± 0.53	105.00 ± 0.39^b,c^	0.000**
DBP (mmHg)	72.92 ± 0.36	85.68 ± 0.40	63.75 ± 0.31^b,c^	0.000**
**Anthropometry**				
Height (cm)	158.12 ± 0.81	159.11 ± 0.60	157.59 ± 0.45	0.542
Weight (Kg)	91.82 ± 0.63	100.10 ± 0.94	49.04 ± 0.83^b,c^	0.000**
BMI (Kg/m^2^)	36.19 ± 0.48	40.01 ± 0.93	19.55 ± 0.53^b,c^	0.000**
MWC (cm)	102.66 ± 0.31	113.96 ± 0.35	73.01 ± 0.33^b,c^	0.000**
Hip C (cm)	123.17 ± 0.83	119.19 ± 0.54	85.30 ± 0.32^b,c^	0.000**
WHR (cm/cm)	0.66 ± 0.02	0.653 ± 0.03	0.57 ± 0.07^b,c^	0.045*
Neck cir. (cm)	39.59 ± 0.79	38.01 ± 0.67	32.62 ± 0.53^b,c^	0.011**
**Skin fold thickness**				
TSF (mm)	31.71 ± 0.12	33.00 ± 0.17	17.30 ± 0.15^b,c^	0.000***
BSF (mm)	26.24 ± 0.13^a^	30.39 ± 0.17	13.72 ± 0.10^b,c^	0.000***
Subscap.(mm)	31.41 ± 0.16	34.32 ± 0.19	14.40 ± 0.11^b,c^	0.000***
Supra-iliac (mm)	27.71 ± 0.26^a^	32.57 ± 0.35	15.80 ± 0.23^b,c^	0.000***
Abd. SF (mm)	31.40 ± 0.15	37.67 ± 0.16	21.10 ± 0.13^b,c^	0.000***
**Body composition**				
Body fat (%)	44.27 ± 0.41	45.96 ± 0.43	23.52 ± 0.33^b,c^	0.000***
FFM (kg)	53.67 ± 0.49	49.93 ± 0.52	37.43 ± 0.37^b,c^	0.000***
Body water (%)	36.55 ± 0.61	39.29 ± 0.66	27.41 ± 0.72^b,c^	0.000**
BMR (KJ)	6538.21 ± 0.18	7034.13 ± 0.17	48,014.31 ± 0.14^b,c^	0.000**

BMI, Body Mass Index; BMR, Basal Metabolic Rate; MWC, Minimal Waist Circumference; TSF, Triceps slin fold; BSF; Biceps skin fold; SBP, Systolic Blood Pressure; DBP, Diastolic Blood Pressure.

*Significant at *p* ≤ 0.05, **highly Significant at *p* ≤ 0.01. *** very highly Significant at *p* ≤  0.001.

^a^Obese without MetS V Obese with Mets, ^b^Obese without MetS V Control, ^c^Obese with Mets V Control.

Table 2 showed the mean ± SE of biochemical parameters of the three studied groups. The obese women with MetS had the significant highest values for the liver enzymes [Aspartate aminotransferase (AST) and the Alanine aminotransferase (ALT)], fasting blood glucose, insulin, and serum lipid profile; except the HDL-C which showed the insignificant lowest value compared to both the obese women without MetS and the control. The mean value of the C-reactive protein had the highly significant highest value among the obese women without MetS. The mean serum concentration of leptin hormone in the obese women with Mets was the significantly lowest compared to the other two groups. SCFA was insignificantly the highest among the obese women with MetS.

**Table 2:** Mean ± SE of biochemical parameters of the three studied groups.

Parameters	Obese No:59	Obese with Mets No:23	Control No:33	*p*
	Mean ± SE			
ALT (U/L)	18.58 ± 0.62	23.26 ± 0.93	15.480.79^c^	0.005**
AST(U/L)	21.03 ± 0.44	25.69 ± 0.35	19.12 ± 0.87^c^	0.001***
Leptin	216.82 ± 0.42	191.18 ± 0.31^a^	253.30 ± 0.47^b.c^	0.017*
SCFA	14.84 ± 0.04	17.47 ± 0.06	12.21 ± 0.01^c^	0.063
CRP	723.42 ± 0.65	693.35 ± 0.74^a^	390.85 ± 0.79^b,c^	0.000***
FBG (mg/dL)	114.66 ± 0.03	130.13 ± 0.34^a^	86.33 ± .27^b,c^	0.000***
Insulin (mIU/L)	12.21 ± 0.07	16.73 ± 0.04	7.93 ± 0.03^b,c^	0.001***
**Lipid profile**				
T C (mg/dL)	195.57 ± 0.45	208.52 ± 0.39^a^	177.82 ± 0.41^b,c^	0.020*
TG (mg/dL)	98.50 ± 0.42	154.39 ± 0.91^a^	73.12 ± 0.71^b,c^	0.000***
HDL-C (mg/dL)	57.01 ± 0.40	54.34 ± 0.64	59.12 ± 0.36	0.332
LDL-C (mg/dL)	117.55 ± 0.51	123.26 ± 0.43	100.73 ± 0.31^b,c^	0.033*

Significant between groups *p ≤ 0.05, **p ≤ 0.01, ***p ≤ 0.001.

FBG, Fasting blood glucose; TC, Total Cholesterol; TG, Triglyceride; HDL-C, High density lipoprotein –Cholesterol LDL-C, Low density lipoprotein-Cholesterol; ALT,Alanine aminotransferase ; AST, Aspartate aminotransferase; SCFA, Short chain fatty acid; CRP, C-reactive protein.

^a^Obese without MetS V Obese with Mets, ^b^Obese without MetS V Control, ^c^Obese with Mets V Control.

Table 3 showed the mean ± SE of the log number and types of Microbiota among the three studied groups. Bacteroidetes bacteria were the most prevalent type among them, followed by the bad gut microbes Firmicutes, while Lactobacillus and Bifidobacteria were the least prevalent. There were significant differences between the log numbers of the 4 types of studied microbiota in the three groups at *p* ≤ 0.021, 0.020, 0.031 respectively. However, insignificant difference was found between the three studied groups, as regard the Firmicutes/Bacteroidetes Ratio (0.72 ± 0.02, 0.69 ± 0.03and 0.73 ± 0.02 respectively) and the two beneficial; the Lactobacillus and Bifidobacteria.

**Table 3:** Mean ± SE of the number and types of Microbiota among the three groups.

Type of microbiota	Obese No:59	Obese with Mets No:23	Control No:33	*p*
	Mean ± SE			
Log Lactobasillus	6.1096 ± 0.096	5.8462 ± 0.174	5.9163 ± 0.068	0.207
Log Bifidobacterium	6.1352 ± 0.091	6.1200 ± 0.121	5.9403 ± 0.088	0.337
Log Bacteroidetes	13.2459 ± 0.196	13.0548 ± 0.205	12.7845 ± 0.167	0.256
Log Firmicutes	9.4530 ± 0.194	8.9127 ± 0.298	9.2845 ± 0.185	0.281
P	0.021*	0.020*	0.031*	
Firmicutes/Bacteroid Ratio	0.7197 ± 0.016	0.6890 ± 0.028	0.7313 ± 0.018	0.456

*Significant at *p* < 0.05.

Table 4 showed the mean ± SE and % of the recommended daily allowances (RDAs) of nutrients intake of the studied women. The obese women with MetS consumed the significant highest percentage of calories represented by the high intake of proteins, total fats and carbohydrates with the lowest fiber intake compared to the other two groups with significant difference at *p* ≤ 0.05–000. The intake of vitamin A and D, potassium, calcium, zinc and iron was low in all groups compared to the RDAs, and it was the lowest among the obese women with MetS with significant difference; except for iron and zinc where there were insignificant differences. The intake of sodium was within the limits of the RDAs. For the daily calcium intake, significant difference was found between the obese women with and without Mets and the control at *p* ≤ 0.021, while insignificant differences were detected for the other two minerals. Consumption of saturate fatty acid (SFAs) was high compared to the RDAs in all the groups, while the daily intake both the monounsaturated fatty acids (MUFAs) and polyunsaturated fatty acids (PUFAs) was the lowest among the obese women with MetS but with insignificant differences. Level of cholesterol intake was high among obese with and without Mets compared to the control with significant difference at *p* ≤ 0.033.The obese women with and without MetS consumed high fat diet which contributed to 42.29–42.89% of the total caloric intake, significant difference at *p* ≤ 0.05 compared to the control was recorded, with insignificant differences in the protein or carbohydrate intake (Table 5).

**Table 4:** Mean ± SE and % of the RDA of nutrients intake of the studied women.

Nutrient intake	Obese NO:59	Obese with Mets No:23	Control No:33	RDAs	*p*
	Mean ± SEM and % of RDAs				
Energy (Cal)	2253.45 ± 9.41 102.43	2520.13 ± 8.32 114.55	1906.89 ± 6.74^c^ 86.68	2200	0.001***
Protein ( g)	74.76 ± 3.69 149.52	86.04 ± 3.52^a^ 172.08	62.26 ± 4.33^b,c^ 124.52	50	0.000***
Fat (g)	105.89 ± 6.39 137.52	120.09 ± 5.73^a^ 155.96	82.21 ± 8.27^b,c^ 106.77	77	0.000***
Carbohydrate (g)	250.35 ± 4.51 83.45	273.79 ± 6.10 91.26	229.49 ± 8.60^c^ 76.50	300	0.026*
Dietary fiber (g)	17.59 ± 0.34 70.36	15.86 ± 0.41 63.44	21.37 ± 0.58^c^ 85.48	25	0.042*
Vit. A (µg)	498.65 ± 0.07 62.33	476.92 ± 0.13 59.62	638.25 ± 0.02^c^ 79.78	800	0.031*
Vit. D (µg)	2.89 ± 0.09 57.80	2.11 ± 0.05 42.20	3.62 ± 0.07^c^ 72.40	5	0.037*
Sodium (mg)	1506.12 ± 21.11 100.41	1510.30 ± 24.01 100.69	1101.26 ± 31.04^b,c^ 73.42	1500	0.024*
Potassium (mg)	1520.80 ± 16.25 76.04	1357.19 ± 12.30 67.86	1610.69 ± 11.73^c^ 80.53	2000	0.031*
Calcium (mg)	381.06 ± 8.17 47.63	338.14 ± 7.11 42.27	565.04 ± 9.10^b,c^ 70.63	800	0.021*
Iron (mg)	5.22 ± 0.50 65.25	4.87 ± 0.30 60.88	5.93 ± 0.20^c^ 74.13	8	0.051
Zinc (mg)	5.43 ± 1.07 45.25	5.10 ± 1.03 42.50	6.29 ± 1.01^c^ 52.42	12	0.067
Sat. FA (g)	33.71 ± 1.13 13.46	37.89 ± 1.09 13.53	24.55 ± 1.20^c^ 11.59	No more than 7% of Total Calories intake	0.065
MUFs (g)	29.02 ± 0.04 11.59	24.07 ± 0.05 8.60	29.88 ± 1.40^c^ 14.10	12–14% of Total Calories intake	0.079
PUFAs (g)	18.04 ± 0.05 7.20	16.11 ± 0.03 5.75	20.15 ± 0.08^c^ 9.51	6–8% of Total Calories intake	0.063
Cholesterol (mg)	238.30 ± 3.17 119.15	264.73 ± 6.72 132.37	192.42 ± 9.15^b,c^ 96.21	200	0.033*

Significant between groups *p ≤ 0.05, **p ≤ 0.01, ***p ≤ 0.001.

^a^Obese without MetS V Obese with Mets, ^b^Obese without MetS V Control, ^c^Obese with Mets V Control.

**Table 5:** The percent of Calories delivered from fat, protein, and carbohydrates of total daily calories intakeof the studied women.

Parameter	Obese without MetS No:59	Obese with MetSNo:23	Control No:33	*p*
	Percent			
Fat	42.29	42.89	38.80	0.047
Protein	13.27	13.65	13.06	0.218
Carbohydrates	44.44	43.46	48.14	0.054

Significance difference *p* ≤ 0.05.

Table 6 showed the Pearson’s correlation coefficient between Firmicutes/Bacteroid Ratio and markers of MetS and other Biochemical parameters of the studied sample. Among the control group, Firmicutes/Bacteroid Ratio had significant positive correlations with SBP, WC, leptin and HDL-C, and significant negative correlations with AST, ALT, insulin, triglycerides and total cholesterol. These correlations became significantly negative with the waist circumference (WC), while significant positive correlation appeared with CRP among the obese group without MetS. Among obese group with MetS, Firmicutes/Bacteroid Ratio had significant positive correlations with total cholesterol and LDL-C and significant negative correlation with SCFA.

**Table 6:** Pearson’s correlation coefficient between Firmicutes/Bacteroid ratio and markers of MetS and other biochemical parameters of the studied sample.

Parameters	Firmicutes/bacteroid ratio					
	Obese without MetS No:59		Obese with MetS No:23		Control No:33	
	R	*p*	r	*p*	r	*p*
SBP	− 0.151	0.284	− 0.391	0.072	0.511	0.011*
DBP	− 0.079	0.579	− 0.103	0.647	0.213	0.318
BMI	0.002	0.990	− 0.231	0.289	0.101	0.595
MWC	− 0.272	0.037*	− 0.145	0.51	0.443	0.014*
% Fat	0.009	0.948	− 0.158	0.471	− 0.295	0.114
AST	− 0.038	0.788	− 0.005	0.981	− 0.606	0.000**
ALT	− 0.028	0.840	− 0.296	0.17	− 0.619	0.000**
Leptin	− 0.061	0.663	− 0.088	0.691	0.530**	0.001**
SCFA	0.029	0.837	− 0.411	0.050*	− 0.253	0.155
BCAA	0.027	0.851	− 0.103	0.64	0.002	0.993
CRP	0.273	0.048*	− 0.301	0.163	− 0.055	0.759
FBG	− 0.218	0.116	− 0.097	0.658	− 0.326	0.064
Insulin	− 0.019 −	0.892	− 0.374	0.079	− 0.439	0.011*
TG	0.007	0.963	0.3	0.164	− 0.362	0.039*
HDL-C	0.233	0.093	− 0.222	0.308	0.461	0.007**
LDL-C	− 0.048	0.734	0.485	0.019*	− 0.342	0.051
TC	− 0.071	0.614	0.562	0.005**	− 0.426	0.014*

Significance difference *p ≤ 0.05, **p ≤ 0.01, ***p ≤ 0.000.

FBG, Fasting blood glucose; TC, Total Cholesterol; TG, Triglyceride; HDL-C, High density lipoprotein –Cholesterol LDL-C, Low density lipoprotein-Cholesterol; ALT, Alanine aminotransferase; AST, Aspartate aminotransferase; SCFA, Short chain fatty acid; CRP, C-reactive protein.

Table 7 showed correlation coefficient between gut microbiota and daily intake of total fat, carbohydrate and fiber among the studied groups. Log Bacteroidetes showed significant positive correlation with the daily fat intake and highly significant negative correlations with the carbohydrate and fiber intake in the control group. Log Lactobacillus had significant positive correlations with the carbohydrate and fiber intake among obese women without MetS. While, Log Bifidobacteria, Log Firmicutes and log Firmicutes/Bacteroid Ratio had insignificant correlations with all the daily intake of total fat, carbohydrate and fiber among the three groups.

**Table 7:** Pearson’s correlation coefficient between gut microbiota and daily intake of total fat, carbohydrate and fiber of the studied groups.

	Log Lactobasillus		Log Bifidobacterium		Log Bacteroidetes		Log Firmicutes		Log Firmicutes/Bacteroid ratio	
	r	*p*	R	*p*	R	*p*	r	*p*	r	*p*
**Obese without Mets**										
Fat	0.064	0.631	− 0.002	0.990	0.090	0.499	− 0.100	0.449	− 0.163	0.217
Carb	0.263	0.045*	− 0.080	0.547	0.098	0.461	0.002	0.991	− 0.072	0.585
Fiber	0.282	0.031*	− 0.029	0.826	0.099	0.454	0.081	0.540	− 0.008	0.951
**Obese with Mets**										
Fat	0.025	0.908	− 0.234	0.282	− 0.095	0.666	0.004	0.987	0.037	0.887
Carb	0.112	0.609	− 0.235	0.279	0.015	0.945	0.093	0.672	0.088	0.680
Fiber	0.140	0.525	− 0.148	0.500	0.088	0.756	− 0.168	0.442	− 0.167	0.447
**Control**										
Fat	0.045	0.402	− 0.279	0.058	0.312	0.038*	− 0.132	0.231	− 0.265	0.068
Carb	− 0.108	0.275	− 0.192	0.142	− 0.462	0.003**	− 0.138	0.223	0.097	0.295
Fiber	− 0.228	0.101	− 0.179	0.159	− 0.480	0.002**	− 0.180	0.158	0.072	0.345

Significance difference *p ≤ 0.05, **p ≤ 0.01.

## Discussion

Obesity has been a motivating force behind the raised medical interest in identification of MetS pathogenesis in western communities and, increasingly, in eastern countries^21^.

Current data of this study reported by dietary history revealed that the obese participated women with MetS consumed the highest percentage of calories represented by the high daily intake of total fats and carbohydrates with lower fiber intake compared to the other two groups. This style of feeding was reflected in the anthropological scales and biochemical parameters, as all of their measures were the highest among the three groups, which is evident in body mass index, waist circumference, as well as all the skin fold thicknesses, in addition to the obvious hyperglycemia, dyslipidemia and higher insulin and CRP concentrations. Oda^22^, and Battault et al.^23^ reported that MetS involved a set of risk factors including obesity, hypertension, hyperglycemia, dyslipideamia, hyperuricemia and others. Uncontrolled MetS will eventually lead to non-alcoholic fatty liver (NAFLD), obstructive sleep apnea-hypo apnea syndrome (OSAHS), and other diseases. The pathogenesis of MetS is correlated with multiple factors, such as chronic inflammation, insulin resistance, oxidative stress and autonomic dysfunction.

### Gut microbiota count

Recently, the disturbance of gut microbiota has been discovered as a risk factor for MetS development^1^. The gut microbiota has developed a symbiotic relationship with the host involving the control of gene expression, gut barrier function, metabolism, nutrition and the general immunological function of the host^24^. Obesity is connected with changes in the relative abundance of the two dominant bacterial divisions; the Bacteroidetes and the Firmicutes; according to Xiao and Kang^25^.

The results of this study are in agreement with what was previously reported where significant increase in Bacteroidetes and the Firmicutes bacteria over the other types (Lactobacillus and Bifido bacteria) was detected. However, interestingly, the data in the current study detected insignificant difference between the three groups for each type of the studied of microbiota. It is likely that the eating pattern of the control lean group was relatively high in fibers which are important for the growth of the beneficial microbiota. Holscher^26^ stated that dietary fibers promote a healthy gut microbome, and that the consumption of dietary fibers and probiotic can modulate the microbiota in the gastrointestinal tract.

### Firmicutes/bacteroidetes ratio and markers of MetS and diet

Obese animals and humans have been found by de Wit et al.^27^ to possess a higher Firmicutes/Bacteroidetes ratio in their gut microbiota than normal-weight people, proposing that this ratio could be used as an obesity biomarker. As a result, the Firmicutes/Bacteroidetes ratio is recently acknowledged as a hallmark of obesity in the scientific literature of Magne et al.^28^. Furthermore, the F/B ratio has been proposed as an important sign of the gut microbiota health^29^. This ratio has been connected to multiple clinical conditions, including those related to ageing^30^ and others associated with obesity and metabolic syndrome^31^. Several studies have explored the link between diet and the gut microbiota because of the potential of dietary interventions to shape the composition of the gut microbiota. Each type of macronutrients (proteins, dietary fibers, fat) influences the gut microbiota specifically. Changes are observed more at a metabolic level than at a taxonomic level with a quick change in gene expression depending on the macronutrients^32^. Gut dysbiosis is associated with various pathologic conditions affecting the gastrointestinal tract (diarrhea, irritable bowel syndrome)^33^, inflammatory bowel diseases^34^, metabolism of the host (obesity, type 2 diabetes, atherosclerosis)^35^.

However, there are some controversies regarding the composition of the gut microbial communities in obese individuals in different populations where several studies have shown contradictory results (for example, reduced F/B ratios in obese individuals^36,37^. There are various reasons for these inconsistent results. Perhaps the association between F/B ratios and obesity depends on specific population, age group, gender, environmental and genetic factors^38^, and as already mentioned, other phyla (Proteobacteria, etc.) has an important role. In addition, the number of specific bacterial species from the Firmicutes and Bactericides phylum associated with obesity is limited. Firmicutes R. bromii is associated with obesity, utilizes and degrades more resistant starch than Eubacteriumrectale, B taiotaomicron, and Bifidobacterium adolescentis^38^. Interestingly, certain Bacteroides species also carry various genes for carbohydrate-degrading enzymes^39^.

In this context, the current study showed low Firmicutes/Bacteroidetes Ratio with only numerical difference between the three studied groups, the detected values were 0.72 and 0.69 inthe obese without and with MetS respectively compared to the control, 0.73. The lack to detect modest differences between healthy and obese subjects was in agreement with Magne et al.^28^ who suggested that the Firmicutes/Bacteroidetes ratio is not a robust marker of micro-biomedysbiosis associated with obesity. Because all the participants in this study belong to the same human race, to similar socio economic level and live in somewhat similar environment, accordingly there was a similarity in their dietary habits. Rinninella et al.^40^ reported that dietary habits can strongly influence gut microbiota composition. Food components have a key impact on the gut microbiota, influencing its composition in terms of richness and diversity^41^

However, data revealed significant correlation between the F/B ratio and some markers of the metabolic syndrome represented by negative correlation with the waist circumference and positive correlation with C-reactive protein in the obese women without MetS, while significant positive correlations were detected with LDL-C and total cholesterol among obese women with MetS. Among the control group, F/B ratio had significant positive correlations with SBP, WC, leptin and HDL-C, and significant negative correlations with AST, ALT, insulin, triglycerides and total cholesterol. This may clarified the presence of changes in the relations between F/B ratio and the different markers of MetS among the obese and normal weight women. This has been shown in results that revealed the difference in the significant response between the biochemical parameters and the Firmicutes/Bacteroid Ratio in the control group. In the same time only few significant relations were detected among obese and MetS women. On this basis, this study indicates the importance of this ratio among Egyptian obese women with and without MetS.

The Present study showed significant positive correlation between Log Bacteroidetes with the daily fat intake, and highly significant negative correlations with the carbohydrate and fiber intake in the control group. However, Log Lactobacillus had significant positive correlations with the carbohydrate and fiber intake among obese women without MetS. On the other hand, Log Bifidobacteria, Log Firmicutes and log Firmicutes/Bacteroidetes ratio had insignificant correlations with the entire daily intake of total fat, carbohydrate and fiber among the three groups.

Diet is one of the provocative factors in progression of obesity and is greatly linked to gut microbiota composition^42^. Nutrient intake and eating habits directly impact the composition, diversity, and metabolism of gut microbiota^42,43^. As previously mentioned, dietary intake of both obese groups revealed high caloric intake with high fat intake and low fiber content. Many dietary patterns such as Western diet, Mediterranean diet, vegetarian diet, and the gluten-free diet have been shown to affect the discrete diversity of the gut microbiota which can disturb host metabolism^43^. The Western diet involves high intake of saturated fats, sugar, salt, refined grains and high fructose corn syrup with a low intake of fibers; it is highly related to obesity and metabolic diseases. The Western diet was found to promote inflammation and changes the profile of the gut microbiota from healthy to the obese pattern^44^. It also has been shown to decrease the total bacteria amount as well as the beneficial Lactobacillus species (sp.) and Bifidobacterium sp. in the gut^45^.

### Short chain fatty acids

The gut microbiota is involved in the development of obesity by direct interactions with proximal organs or indirect interactions with distant organs through metabolic products (mainly SCFAs) including communication with the liver, adipose tissue, and brain^46^. SCFAs can play a crucial role in the pathogenesis of obesity. They interact with adipose tissue via two G-protein-coupled receptors expressed in adipocytes (Gpr41 and Gpr43); this promotes adipocytes formation and inhibits lipolysis^47^. Furthermore, SCFAs down regulate the synthesis of the hunger-suppressing hormones leptin, peptide YY, and glucagon-like peptide 1^48^. SCFA influence lipid and glucose metabolism in order to provide energy for the host, proposing that they may have an impact on the occurrence of metabolic risk factors^49^.

Data of this study showed that the serum concentration of SCFA was mostly elevated among the obese women with MetS and in the same time showed negative association with the Firmicutes/Bacteroidetes ratio. Also it was evident that the levels of liver enzymes and the serum insulin concentration were also the highest among obese MetS participants, while the level of the leptin hormone was the lowest, which agree with what was mentioned in the previous research.

## Conclusion

It was concluded from this study that both Bacteroidetes and Firmicutes bacteria are the most abundant bacteria in the gut among the studied sample, whether in the obese with and without MetS or normal weights women. In addition the results confirm the low Firmicutes/Bacteroid Ratio among all groups. No clear relations were found between this ratio and the fat, carbohydrate, fiber content of the diets. It was one of the most important findings of this research: the links between the short chain fatty acids which is the metabolic product of the gut microbiota and the promotion of obesity and the ensuing metabolic disorders.

## Data Availability

The datasets generated and/or analyzed during the current study are not publicly available [due restrictions from our institute “National Research Centre”; which funded this study as a part of our jobs], but are available from the corresponding author on reasonable request, after taking the permission of our institute “National Research Centre”.

## References

[1] Agus A, Clément K, Sokol H (2021). Gutmicrobiota-derived metabolites as central regulators in metabolic disorders. Gut.

[2] Saklayen MG (2018). The global epidemic of the metabolic syndrome. Curr. Hypertens. Rep..

[3] Kim MH, Yun KE, Kim J, Park E, Chang Y, Ryu S, Kim HL, Kim HN (2020). Gut microbiota and metabolic health among overweight and obese individuals. Sci. Rep..

[4] Wang PX, Deng XR, Zhang C, Yuan HJ (2020). Gut microbiota and metabolic syndrome. Chin. Med. J. (Engl.).

[5] Sender R, Fuchs S, Milo R (2016). Revised estimates for the number of human and bacteria cells in the body. PLoS Biol..

[6] Thursby E, Juge N (2017). Introduction to the human gut microbiota. Biochem. J..

[7] Zheng D, Liwinski T, Elinav E (2020). Interaction between microbiota and immunity in health and disease. Cell Res..

[8] Rinninella E, Raoul P, Cintoni M, Franceschi F, Miggiano GAD, Gasbarrini A, Mele MC (2019). What is the healthy gut microbiota composition? A changing ecosystem across age, environment, diet, and diseases. Microorganisms.

[9] Stojanov S, Berlec A, Štrukelj B (2020). The influence of probiotics on the firmicutes/bacteroidetes ratio in the treatment of obesity and inflammatory bowel disease. Microorganisms.

[10] Zaky A, Glastras SJ, Wong MYW, Pollock CA, Saad S (2021). The role of the gut microbiome in diabetes and obesity-related kidney disease. Int. J. Mol. Sci..

[11] Rowland I, Gibson G, Heinken A, Scott K, Swann J, Thiele I, Tuohy K (2018). Gut microbiota functions: Metabolism of nutrients and other food components. Eur. J. Nutr..

[12] Blaak EE, Canfora EE, Theis S, Frost G, Groen AK, Mithieux G, Nauta A, Scott K, Stahl B, van Harsselaar J, van Tol R, Vaughan EE, Verbeke K (2020). Short chain fatty acids in human gut and metabolic health. Benef. Microbes.

[13] Hiernaux J, Tanner J, Weiner JS, Lourie SA (1969). Growth and physical studies. Human Biology: Aguide to Field Methods.

[14] World Food Dietary Assessment System (1995). WFDAS.

[15] Considine RV, Sinha MK, Heiman ML, Kriauciunas A, Stephens TW, Nyce MR, Ohannesian JP, Marco CC, McKee LJ, Bauer TL (1996). Serum immunoreactive-leptin concentrations in normal-weight and obese humans. N. Engl. J. Med..

[16] denBesten G, van Eunen K, Groen AK, Venema K, Reijngoud DJ, Bakker BM (2013). The role of short-chain fatty acids in the interplay between diet, gut microbiota, and host energy metabolism. J. Lipid. Res..

[17] Mitra B, Panja M (2005). High sensitive C-reactive protein: A novel biochemical markers and its role in coronary artery disease. J. Assoc. Phys. India.

[18] Friedewald WT, Levy RI, Fredrickson DS (1972). Estimation of the concentration of low-density lipoprotein cholesterol in plasma, without use of the preparative ultracentrifuge. Clin. Chem..

[19] Alberti KG, Eckel RH, Grundy SM, Zimmet PZ, Cleeman JI, Donato KA, Fruchart JC, James WP, Loria CM, Smith SC (2009). International Diabetes Federation Task Force on Epidemiology and Prevention; Hational Heart, Lung, and Blood Institute; American Heart Association; World Heart Federation; International Atherosclerosis Society; International Association for the Study of Obesity. Harmonizing the metabolic syndrome: a joint interim statement of the International Diabetes Federation Task Force on Epidemiology and Prevention; National Heart, Lung, and Blood Institute; American Heart Association; World Heart Federation; International Atherosclerosis Society; and International Association for the Study of Obesity. Circulation.

[20] Wheeler HE, Shah KP, Brenner J, Garcia T, Aquino-Michaels K, Cox NJ, Nicolae DL, Im HK, GTEx Consortium (2016). Survey of the heritability and sparse architecture of gene expression traits across human tissues. PLoS Genet..

[21] Belete R, Ataro Z, Abdu A, Sheleme M (2021). Global prevalence of metabolic syndrome among patients with type I diabetes mellitus: A systematic review and meta-analysis. Diabetol. Metab. Syndr..

[22] Oda E (2018). Historical perspectives of the metabolic syndrome. Clin. Dermatol..

[23] Battault S, Meziat C, Nascimento A, Braud L, Gayrard S, Legros C (2018). Vascular endothelial function masks increased sympathetic vasopressor activity in rats with metabolic syndrome. Am. J. Physiol. Heart Circ. Physiol..

[24] Liu R, Hong J, Xu X, Feng Q, Zhang D, Gu Y (2017). Gut microbiome and serum metabolome alterations in obesity and after weight-loss intervention. Nat. Med..

[25] Xiao H, Kang S (2020). The role of the gut microbiome in energy balance with a focus on the gut-adipose tissue axis. Front. Genet..

[26] Holscher HD (2017). Dietary fiber and prebiotics and the gastrointestinal microbiota. Gut Microbes.

[27] de Wit N, Derrien M, Bosch-Vermeulen H, Oosterink E, Keshtkar S, Duval C, de Vogel-van den Bosch J, Kleerebezem M, Müller M, van der Meer R (2012). Saturated fat stimulates obesity and hepatic steatosis and affects gut microbiota composition by an enhanced overflow of dietary fat to the distal intestine. Am. J. Physiol. Liver Physiol..

[28] Magne F, Gotteland M, Gauthier L, Zazueta A, Pesoa S, Navarrete P, Balamurugan R (2020). The Firmicutes/Bacteroidetes ratio: A relevant marker of gut dysbiosis in obese patients?. Nutrients.

[29] Li W, Ma ZS (2020). FBA ecological guild: Trio of Firmicutes-Bacteroidetes alliance against Actinobacteria in human oral microbiome. Sci. Rep..

[30] Liang D, Leung RK, Guan W (2018). Involvement of gut microbiome in human health and disease: Brief overview, knowledge gaps and research opportunities. Gut Pathog..

[31] Woting A, Blaut M (2016). The intestinal microbiota in metabolic disease. Nutrients.

[32] Aguirre M, Eck A, Koenen ME, Savelkoul PHM, Budding AE, Venema K (2016). Diet drives quick changes in the metabolic activity and composition of human gut microbiota in a validated in vitro gut model. Res. Microbiol..

[33] Carding S, Verbeke K, Vipond DT, Corfe BM, Owen LJ (2015). Dysbiosis of the gut microbiota in disease. Microb. Ecol. Health Dis..

[34] Hills RD, Pontefract BA, Mishcon HR, Black CA, Sutton SC, Theberge CR (2019). Gut microbiome profound. Implications for diet and disease. Nutrients.

[35] Yong VB (2017). The role of the microbiome in human health and disease. An introduction for clinicians. BMJ.

[36] Castaner O, Goday A, Park YM, Lee SH, Magkos F, Shiow STE, Schröder H (2018). The gut microbiome profile in obesity: A systematic review. Int J Endocrinol..

[37] Vaiserman A, Romanenko M, Piven L, Moseiko V, Lushchak O, Kryzhanovska N, Guryanov V, Koliada A (2020). Differences in the gut Firmicutes to Bacteroidetes ratio across age groups in healthy Ukrainian population. BMC Microbiol..

[38] Ze X, Duncan SH, Louis P, Flint HJ (2012). Ruminococcusbromii is a keystone species for the degradation of resistant starch in the human colon. ISME J..

[39] Flint HJ, Scott KP, Duncan SH, Louis P, Forano E (2012). Microbial degradation of complex carbohydrates in the gut. Gut Microbes.

[40] Rinninella E, Cintoni M, Raoul P, Lopetuso LR, Scaldaferri F, Pulcini G, Miggiano GAD, Gasbarrini A, Mele MC (2019). Food components and dietary habits: Keys for a healthy gut microbiota composition. Nutrients.

[41] Mayer EA, Tillisch K, Gupta A (2015). Gut/brain axis and the microbiota. J. Clin. Investig..

[42] Brahe LK, Astrup A, Larsen LH (2016). Can we prevent obesity-related metabolic diseases by dietary modulation of the gut microbiota?. Adv. Nutr..

[43] Lazar V, Ditu LM, Pircalabioru GG, Picu A, Petcu L, Cucu N (2019). Gut microbiota, host organism, and diet trialogue in diabetes and obesity. Front. Nutr..

[44] Statovci D, Aguilera M, MacSharry J, Melgar S (2017). The impact of Western diet and nutrients on the microbiota and immune response at mucosal interfaces. Front. Immunol..

[45] Bell DS (2015). Changes seen in gut bacteria content and distribution with obesity: Causation or association?. Postgrad. Med..

[46] Mitev K, Taleski V (2019). Association between the gut microbiota and obesity. Open Access Maced. J. Med. Sci..

[47] Kimura I, Ozawa K, Inoue D, Imamura T, Kimura K, Maeda T, Terasawa K, Kashihara D, Hirano K, Tani T, Takahashi T, Miyauchi S, Shioi G, Inoue H, Tsujimoto G (2013). The gut microbiota suppresses insulin-mediated fat accumulation via the short-chain fatty acid receptor GPR43. Nat. Commun..

[48] Tseng CH, Wu CY (2019). The gut microbiome in obesity. J. Formos. Med. Assoc..

[49] Teixeira TF, Grześkowiak Ł, Franceschini SC, Bressan J, Ferreira CL, Peluzio MC (2013). Higher level of faecal SCFA in women correlates with metabolic syndrome risk factors. Br. J. Nutr..

